# Exogenous Hydrogen Sulfide Plays an Important Role Through Regulating Autophagy in Ischemia/Reperfusion Injury

**DOI:** 10.3389/fmolb.2021.681676

**Published:** 2021-05-13

**Authors:** Shuangyu Lv, Zhu Wang, Jie Wang, Honggang Wang

**Affiliations:** ^1^Henan International Joint Laboratory for Nuclear Protein Regulation, School of Basic Medical Sciences, Henan University, Kaifeng, China; ^2^Henan Technician College of Medicine and Health, Kaifeng, China

**Keywords:** hydrogen sulfide, autophagy, ischemia/reperfusion injury, apoptosis, oxidative stress

## Abstract

Ischemia/reperfusion (I/R) injury is characterized by limiting blood supply to organs, then restoring blood flow and reoxygenation. It leads to many diseases, including acute kidney injury, myocardial infarction, circulatory arrest, ischemic stroke, trauma, and sickle cell disease. Autophagy is an important and conserved cellular pathway, in which cells transfer the cytoplasmic contents to lysosomes for degradation. It plays an important role in maintaining the balance of cell synthesis, decomposition and reuse, and participates in a variety of physiological and pathological processes. Hydrogen sulfide (H_2_S), along with carbon monoxide (CO) and nitric oxide (NO), is an important gas signal molecule and regulates various physiological and pathological processes. In recent years, there are many studies on the improvement of I/R injury by H_2_S through regulating autophagy, but the related mechanisms are not completely clear. Therefore, we summarize the related research in the above aspects to provide theoretical reference for future in-depth research.

## Introduction

**FIGURE 1:**
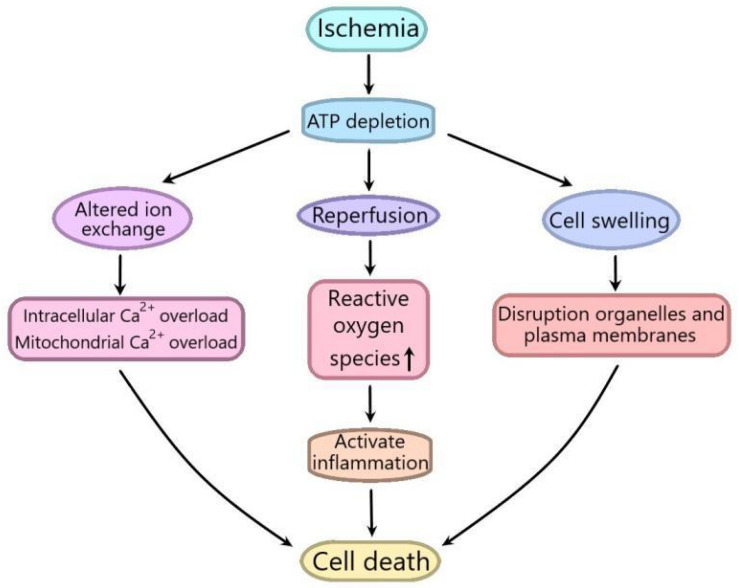
Schematic diagram of the mechanism of ischemia/reperfusion (I/R) injury. Ischemia increases intracellular and mitochondrial calcium levels by interfering with ATPase dependent ion transport. At the same time, the mechanism of cell volume regulation is also destroyed by the lack of ATP, which can cause the dissolution of organelles and plasma membranes. Although reperfusion can save hypoxic tissue, it can promote the production of reactive oxygen species, isolate pro-inflammatory immune cells in ischemic tissue, and aggravate tissue injury.

Ischemia/reperfusion (I/R) injury is a term used to represent the functional and structural changes that become obvious during the recovery of blood flow after a period of ischemia. In addition to ischemia, the recovery of blood flow can also lead to potentially very harmful effects, including significant cell swelling, irreversible cell necrosis and uneven blood flow recovery in all parts of the tissue (Peralta et al., 2013; Soares et al., 2019). I/R injury is composed of two important events. The first one refers to the limitation of blood supply to an organ, usually due to the blockage of blood supply in the artery by an embolus. Ischemic are always associated with cell metabolic imbalance and harmful hypoxia. The second one is reperfusion, which may lead to a destructive inflammatory response to further aggravate tissue damage (Yellon and Hausenloy, 2007; Eltzschig and Eckle, 2011). The degree of tissue damage is directly to the degree of blood reduction and the length of ischemia time. Ischemia increases intracellular and mitochondrial calcium levels through disturbing ATPase dependent ion transport. At the same time, the mechanism of cell volume regulation is also destroyed by the lack of ATP, which can induce the dissolution of organelles and plasma membranes. Reperfusion, although rescuing oxygen-starved tissues, promotes the production of reactive oxygen species, isolates the proinflammatory immunocytes in ischemic tissues, and aggravates tissue damage (Kalogeris et al., 2017; Figure 1). I/R injury leads to a lot of serious clinical problems in many pathological processes including acute myocardial infarction, kidney injury (AKI), ischemic stroke, and circulatory arrest (Tang and Zhuang, 2019). Although the roles of I/R injury in many organs have been widely studied, including heart, brain and kidney, the related exact molecular mechanisms are not fully understood (Yellon and Hausenloy, 2007; Hanson et al., 2009; Angeli et al., 2014).

Autophagy is an evolutionarily conserved process in which the cytoplasm and organelles are isolated in double membrane vesicles, then transported to lysosomes/vacuoles for degradation and recycling of eukaryotic cytoplasmic components (Galluzzi et al., 2017; Guo et al., 2019). According to the differences in cargo specificity and delivery to lysosomes, autophagy is divided into three types: macroautophagy, microautophagy and chaperone-mediated autophagy (Gomes et al., 2017). In macroautophagy (also known as autophagy), cytoplasmic components are engulfed in double membranous vesicles to form autophagosomes. Autophagosomes then fuse with lysosomes to form autolysosomes in which cargoes are degraded or recycled (Mizushima and Komatsu, 2011; Galluzzi et al., 2017; Bonam et al., 2019); Microautophagy refers to the direct invagination of lysosomal membrane and encapsulation of cell contents (Mijaljica et al., 2011); Chaperone-mediated autophagy is a kind of selective autophagy, in which proteins in cells are binded with chaperones, then transported to lysosomal chambers for deradation (Fujiwara et al., 2017; Kaushik and Cuervo, 2018). Autophagy plays an important role in organ homeostasis, anti-aging mechanism, and immune response (Mizushima et al., 2011; Deretic et al., 2013; Kaushik and Cuervo, 2015). Autophagy can be induced by hypoxia, ischemia, pathogen infection, protein misfolding, hormone therapy, nutritional deficiency, and other internal and external factors (Matsui et al., 2007; Ravanan et al., 2017). Abnormal autophagy plays an important role in many pathological processes including ischemia-reperfusion injury (Wang et al., 2019), inflammatory and infectious diseases, obesity and type 2 diabetes, cancer, and neurodegenerative diseases (Choi et al., 2013; Jiang and Mizushima, 2014). The relevant mechanism has not been fully understood.

Hydrogen sulfide (H_2_S) has always been considered as a toxic pollutant, but recently it, together with nitric oxide (NO) and carbon monoxide (CO), is regarded as a biological signal molecule, namely gas transmitter (Szabo, 2007; Powell et al., 2018). To date, it is generally believed that endogenous H_2_S is mainly produced by three enzymes, namely cystathionine-β-synthase (CBS), cystathionine-γ-lyase (CSE), as well as 3-mercaptopyruvate sulfurtransferase (3-MST) (Polhemus and Lefer, 2014). H_2_S has many physiological functions, including anti-inflammatory (Du et al., 2014), anti-apoptotic (Guo et al., 2014), relaxing blood vessels, reducing blood pressure (Yang et al., 2008; Sun et al., 2015) and anti oxidative stress (Zheng et al., 2015). It has been reported that during I/R, the structure of mitochondria is damaged and the production of reactive oxygen species (ROS) increased, H_2_S can protect the integrity of mitochondria and reduce the production of mitochondrial reactive oxygen species (mtROS), thus alleviating I/R injury (Andreadou et al., 2020). In recent years, there are many studies on the effects of H_2_S on autophagy to improve I/R injury, but its mechanism is not fully clear. Therefore, we summarize the relevant research in the above aspects to provide theoretical reference for future in-depth research.

## Exogenous H_2_S Plays an Important Role Through Regulating Autophagy in Hepatic I/R Injury

Hepatic I/R injury usually occurs in the process of shock and liver transplantation, which is an important clinical problem and a serious threat to human health (Dogan and Aslan, 2011). Therefore, how to improve hepatic I/R injury is increasingly important. Hepatic I/R can induce autophagy, and the suppression of autophagy alleviates hepatic I/R injury (Shen et al., 2013). Exogenous H_2_S has been reported to play a protective role against hepatic I/R injury (Kang et al., 2010). In order to investigate the effects of exogenous H_2_S on autophagy in hepatic I/R injury and the related mechanism, Cheng et al. (2014) conducted a series of experiments and found that exogenous H_2_S improved hepatic I/R injury by notably decreasing I/R-induced serum levels of alanine aminotransferase (ALT), aspartate transaminase (AST) and inflammatory cytokines, improving pathological changes of liver and reducing hepatocyte apoptosis. The mechanism research revealed that autophagy was enhanced in I/R-induced hepatitis, which could be reversed by exogenous H_2_S. Moreover, exogenous H_2_S inhibited c-Jun NH2-terminal kinases (JNK) pathway induced by I/R injury through decreasing JNK1 and extracellular signal-regulated kinase (ERK) phosphorylation, while SP600125, a JNK1 inhibitor, strengthened H_2_S hepatoprotective effects, indicating that autophagy and JNK pathway are involved in the hepatoprotective effect of exogenous H_2_S in hepatic I/R injury (Cheng et al., 2014). The evidences indicate that the suppression of JNK activation with SP600125 can block autophagy (Rodriguez-Enriquez et al., 2006), so it can be inferred that exogenous H_2_S improve hepatic I/R injury by inhibiting autophagy through suppressing JNK pathway. Apoptosis interacts with autophagy. The activation of autophagy can inhibit apoptosis, while the inhibition of autophagy can promote cell death and apoptosis (Wang et al., 2012). It has been reported that the upregulation of autophagy can reduce hepatic I/R injury (Cardinal et al., 2009), which is contradicts the above inference that inhibiting autophagy improves hepatic I/R injury. In the above I/R-induced hepatitis, 3-MA, an autophagy inhibitor, further decreased H_2_S-inhibited autophagy, which abolished the protective effect of exogenous H_2_S against I/R-induced hepatic injury. Whereas rapamycin, an autophagy enhancer, had the opposite effect. This seems to contradict the previous inference that exogenous H_2_S improves hepatic I/R injury by inhibiting autophagy (Cheng et al., 2014). The reason may be that the regulation of autophagy by exogenous H_2_S has two-sided effects in liver I/R injury. The moderate inhibition of autophagy by H_2_S has cytoprotective effect, while the excessive inhibition of autophagy shows the opposite result. Scavenger receptor A (SRA) is one of the main receptors involved in macrophage-mediated inflammatory response, which plays an important role in cerebral ischemia injury (Ohnishi et al., 2011; Xu et al., 2012). The activation of SRA has been reported to suppress autophagy in macrophages (Huang et al., 2014). Exogenous H_2_S could improve fatty liver I/R injury through mitigating the pathological changes of liver tissue and decreasing the levels of ALT, AST, and lactate dehydrogenase (LDH). The mechanism research showed that exogenous H_2_S promoted the autophagy of peritoneal macrophages by increasing the concentration of LC3B particles and the ratio of LC3-II/LC3-I in fatty liver I/R injury. Exogenous H_2_S also inhibited inflammation, apoptosis and oxidative stress, and decreased the protein expression of SRA in fatty liver I/R injury, suggesting that exogenous H_2_S alleviated hepatic I/R injury by enhancing autophagy through suppressing SRA pathway, which needed to be further studied by using the related inhibitors (Ruan et al., 2019). SRA pathway may be an important target in the effects of H_2_S on autophagy in hepatic I/R injury.

## Exogenous H_2_S Plays an Important Role Through Regulating Autophagy in Myocardial I/R Injury

Rapid coronary artery reperfusion is a common treatment for myocardial ischemia. However, reperfusion itself can lead to injury, which is called myocardial I/R injury (Chang et al., 2013). Therefore, it is necessary to find a new way to reduce myocardial I/R injury. Myocardial ischemia enhances Adenosine monophosphate -activated protein kinase (AMPK) activation and induces protective autophagy (Matsui et al., 2007), the mechanism of which is not clear. Hong Xie, et al. found that exogenous H_2_S could improve myocardial I/R injury by reducing myocardial infarction. Myocardial I/R induced the formation of autophagosome, impaired the clearance of autophagosome, decreased the levels of p-AMPK/AMPK, LC3-II/I and beclin-1, and increased p62 and LAMP-2 protein levels, while exogenous H_2_S reversed these changes, indicating that exogenous H_2_S promoted autophagy and activated AMPK pathway. Moreover, the AMPK inhibitor Compound C and autophagy inhibitor CQ abolished the cardioprotective effect of exogenous H_2_S on myocardial I/R injury, indicating that exogenous H_2_S improved myocardial I/R injury by promoting AMPK-mediated autophagy impaired by I/R injury (Xie et al., 2015). AMPK-mediated autophagy plays a dual role in the process of myocardial I/R injury. Myocardial ischemia can up- regulate AMPK- mediated autophagy to play a protective role, while subsequent reperfusion has the opposite effect. Contrary to the above conclusion that enhanced autophagy by H_2_S can ameliorate myocardial I/R injury, enhancing autophagy may lead to myocardial cell death through excessive self-digestion and degradation of essential cell components (Matsui et al., 2007; Hariharan et al., 2011). After myocardial hypoxia/reoxygenation (H/R), autophagy increased significantly, while exogenous H_2_S could suppress autophagy to protect myocardium. Blocking phosphatidylinositol-3-kinase (PI3K) with LY294002, a PI3K inhibitor, or knocking down Serum- and glucocorticoid-induced kinase 1 (SGK1) with SGK1 siRNA could enhance autophagy and weakened the anti-autophagy and cardioprotection effects of exogenous H_2_S, while the inhibition of glycogen synthase kinase-3 beta (GSK3β) with tws119, a GSK3β inhibitor, had the opposite effects. Collectively, exogenous H_2_S plays a cardioprotective role in neonatal rat cardiomyocytes exposed to H/R by inhibiting autophagy through PI3K/SGK1/GSK3β signaling pathway (Jiang et al., 2016).

## Exogenous H_2_S Plays an Important Role Through Regulating Autophagy in Cerebral I/R Injury

Cerebral infarction is the leading cause of death and permanent disability of adults all over the world (Yan et al., 2016). The injury of brain cells caused by cerebral ischemia is further aggravated after the recovery of blood supply, which is called cerebral I/R injury (Molina and Alvarez-Sabin, 2009). Autophagy has been reported to be involved in cerebral I/R injury (Fan et al., 2016). The research of Shui et al. (2016) showed that exogenous H_2_S alleviated the cerebral I/R injury through decreasing cortex infarct in middle cerebral artery occlusion (MCAO) mice, while autophagy activator rapamycin alleviated the protective effects of H_2_S, and the autophagy inhibitor 3-MA had the similar results to exogenous H_2_S, suggesting that autophagy was involved in the protective effects of exogenous H_2_S. Exogenous H_2_S suppressed the elevation of LC3-II and the decrease of p62, but had no notably effect on Beclin-1 complex in cerebral I/R injury model mice, indicating that exogenous H_2_S inhibited autophagy through decreasing autophagosome accumulation. From the above, it can be deduced that exogenous H_2_S reduced cerebral cortex I/R injury through inhibiting autophagy (Shui et al., 2016). The decrease of autophagosome accumulation leaded by exogenous H_2_S may be due to the decrease of autophagosome synthesis or the acceleration of autophagosome degradation (Klionsky et al., 2008; Bonam et al., 2020), therefore, in order to determine whether exogenous H_2_S suppresses autophagy by decreasing autophagosome synthesis or increasing autophagosome degradation, Zhu et al. (2017) employed SH-SY5Y cells for the oxygen and glucose deprivation/reoxygenation (OGD/R) and mice for the cerebral I/R to performed *in vitro* and *in vivo* experiments, respectively, and found exogenous H_2_S notably decreased cerebral infarct volume, increased cell viability and downregulated the OGD/R-induced elevation in LC3-II, which was abolished by co-treatment with the autophagy maturation inhibitor bafilomycin A1 (BafA1). Moreover, exogenous H_2_S had no influences on the -induced upregulation of the ULK1 self-association and reduction of the ATG13 phosphorylation induced by OGD/R, which are vital for the initiation of autophagosome formation. Taken together, it can be inferred that exogenous H_2_S played neuroprotection against cerebral I/R injury through increasing autophagosome degradation (Zhu et al., 2017). To further prove the protective effect of H2S on cerebral I/R injury by suppressing autophagy, Jiang et al. (2017) exposed PC12 cells to hypoxia glucose deprivation/reoxygenation (OGD/R) *in vitro* to simulate MCAO and found that exogenous H_2_S alleviated OGD/R-induced cellular injury of PC12 cells by reducing apoptosis and inhibited autophagy induced by OGD/R by reducing the ratio of LC3-II/I and increasing the expression of p62. Rapamycin abolished the protective effects of exogenous H_2_S on OGD/R-induced cellular injury of PC12 cells, and the autophagy inhibitor 3-MA had the similar results to H_2_S, suggesting that exogenous H_2_S improved the cellular injury of PC12 cells induced by OGD/R through inhibiting autophagy (Jiang et al., 2017). Previous study has shown that exogenous H_2_S improves cerebral I/R injury through inhibiting oxidative stress and apoptosis (Yu et al., 2015). Whether exogenous H_2_S alleviates cerebral I/R injury by inhibiting autophagy via suppressing oxidative stress and apoptosis needs to be elucidated.

## Exogenous H_2_S Plays an Important Role Through Regulating Autophagy in Renal I/R Injury

Renal I/R injury is a typical complication of kidney injury after organ transplantation, which leads to accumulation of toxic metabolites, depletion of adenosine triphosphate (ATP), and subsequent tissue injury. In the process of renal transplantation, the transient stop of renal blood flow leads to acute ischemic injury, and the subsequent reperfusion further deepens the functional and structural damage of human kidney (Wang et al., 2004; Wu et al., 2007). At present, it is very urgent to explore the effective prevention of renal I/R injury. Ling et al. (2017) found that in renal I/R injury patients, 24 h after I/R, the levels of IL-6, transforming growth factor-beta (TGF-β), LC3II/I, endoplasmic reticulum stress pathway related genes, apoptotic index and autophagosome number increased significantly, the levels of p62 and SR-A, superoxide dismutase (SOD) and H_2_S concentration decreased significantly, while exogenous H_2_S significantly reversed the changes. SR-A gene knockout had the similar effects to exogenous H_2_S (Ling et al., 2017), Moreover, exogenous H_2_S alleviated hepatic I/R injury by enhancing autophagy through suppressing SRA pathway (Ruan et al., 2019). Therefore, it can be inferred that exogenous H_2_S improved renal I/R injury by inhibiting autophagy through suppressing SRA pathway, which needed further studied. In particularly, the mechanism of exogenous H_2_S regulating SRA pathway remains to be elucidated.

## Exogenous H_2_S Plays an Important Role Through Regulating Autophagy in Spinal Cord I/R Injury

Spinal cord I/R injury is a dynamic process, which often occurs in various clinical conditions, such as thoracoabdominal artery surgery or spinal cord injury. Its pathogenesis is very complex including membrane permeability changes, lipid oxidation and so on. Therefore, it is difficult to achieve the purpose of effective treatment (Ege et al., 2004; Korkmaz et al., 2013). The results of Li et al. (2015) showed that exogenous H_2_S ameliorated motor function and infarct area, promoted autophagy by upregulating the expression levels of beclin-1 and LC3II, and downregulating miR-30c expression in rat spinal cord I/R injury model, indicating that autophagy and miR-30c were involved in exogenous H_2_S improvement of spinal cord I/R injury. Exogenous H_2_S also improved oxygen glucose deprivation (OGD)-induced ischemic injury in SY-SH-5Y cells (Li et al., 2015). It has been reported that miR-30c can regulate autophagy (Wang et al., 2014). The results showed that miR-30c negatively regulated Beclin-1 expression in SY-SH-5Y cells treated with OGD. Moreover, the spinal cord improvement of exogenous H_2_S was reversed by 3-MA and miR-30c overexpression, suggesting that exogenous H_2_S ameliorated spinal cord I/R injury by promoting autophagy via suppressing miR-30c, which might have potential clinical value in the treatment of spinal cord I/R injury (Zhu et al., 2017). The regulation of microRNA on autophagy needs to be further studied.

## Conclusion

In this review, the effects of exogenous H_2_S on autophagy in I/R injury was summarized as follows: (1) Exogenous H_2_S improve hepatic I/R injury by inhibit autophagy through suppressing JNK pathway; (2) Exogenous H_2_S alleviated hepatic I/R injury by enhancing autophagy through suppressing SRA pathway; (3) Exogenous H_2_S improved myocardial I/R injury by promoting AMPK-mediated autophagy or by inhibiting autophagy through PI3K/SGK1/GSK3β pathway; (4) Exogenous H_2_S reduced cerebral I/R injury through inhibiting autophagy via promoting autophagosome degradation; (5) Exogenous H_2_S improved renal I/R injury by inhibiting autophagy through suppressing SRA pathway; and (6) Exogenous H_2_S ameliorated spinal cord I/R injury by promoting autophagy via suppressing miR-30c (Table 1). It can be concluded that exogenous H_2_S can improve ischemia-reperfusion injury by inhibiting autophagy or promoting autophagy. This difference may be due to the different basic levels of autophagy in different tissues, which leads to different responses of autophagy to ischemia-reperfusion injury. In addition, the mechanism of the effects of H_2_S on autophagy and the response mechanism of autophagy to ischemia-reperfusion injury need to be further elucidated.

**TABLE 1:** The effects of hydrogen sulfide (H_2_S) on autophagy in different ischemia/reperfusion (I/R) injury.

The name of disease	State of autophagy	The role of H_2_S
Hepatic I/R injury	Overactivation/Inhibition	Improve hepatic I/R injury via inhibiting/promoting autophagy
Myocardial I/R injury	Inhibition	Improve invocardial I/R injury via promoting autophagy
Cerebral I/R injury	Overactivation	Improve cerebral I/R injury through inhibiting autophagy
Renal I/R injury	Overactivation	Improve renal I/R injury by inhibiting autophagy
Spinal cord I/R injury	Inhibition	Improve spinal cord I/R injury by promoting autophagy

With the development of research, the effect of exogenous H_2_S on autophagy may provide a new strategy for the prevention and treatment of ischemia-reperfusion injury.

## Author Contributions

HW revised, wrote, and funded the manuscript. SL wrote and funded the manuscript. ZW and JW wrote the manuscript. All authors contributed to the article and approved the submitted version.

## Conflict of Interest

The authors declare that the research was conducted in the absence of any commercial or financial relationships that could be construed as a potential conflict of interest.
